# Combined Hyperthermia and Radiotherapy for the Treatment of Cancer

**DOI:** 10.3390/cancers3043799

**Published:** 2011-09-30

**Authors:** Punit Kaur, Mark D. Hurwitz, Sunil Krishnan, Alexzander Asea

**Affiliations:** 1 Department of Pathology, Scott & White Hospital and the Texas A&M Health Science Center, College of Medicine, Temple, TX 76504, USA; E-Mail: pkaur@medicine.tamhsc.edu; 2 Department of Radiation Oncology, Dana-Farber/Brigham and Women's Cancer Center and Harvard Medical School, Boston, MA 02115, USA; E-Mail: mhurwitz@lroc.harvard.edu; 3 Department of Radiation Oncology, The University of Texas MD Anderson Medical Center, Houston, TX 77030, USA; E-Mail: skrishnan@mdanderson.org

**Keywords:** heat shock proteins, radiotherapy, hyperthermia, cancer, hypoxia

## Abstract

Radiotherapy is used to treat approximately 50% of all cancer patients, with varying success. Radiation therapy has become an integral part of modern treatment strategies for many types of cancer in recent decades, but is associated with a risk of long-term adverse effects. Of these side effects, cardiac complications are particularly relevant since they not only adversely affect quality of life but can also be potentially life-threatening. The dose of ionizing radiation that can be given to the tumor is determined by the sensitivity of the surrounding normal tissues. Strategies to improve radiotherapy therefore aim to increase the effect on the tumor or to decrease the effects on normal tissues, which must be achieved without sensitizing the normal tissues in the first approach and without protecting the tumor in the second approach. Hyperthermia is a potent sensitizer of cell killing by ionizing radiation (IR), which can be attributed to the fact that heat is a pleiotropic damaging agent, affecting multiple cell components to varying degrees by altering protein structures, thus influencing the DNA damage response. Hyperthermia induces heat shock protein 70 (Hsp70; HSPA1A) synthesis and enhances telomerase activity. HSPA1A expression is associated with radioresistance. Inactivation of HSPA1A and telomerase increases residual DNA DSBs post IR exposure, which correlates with increased cell killing, supporting the role of HSPA1A and telomerase in IR-induced DNA damage repair. Thus, hyperthermia influences several molecular parameters involved in sensitizing tumor cells to radiation and can enhance the potential of targeted radiotherapy. Therapy-inducible vectors are useful for conditional expression of therapeutic genes in gene therapy, which is based on the control of gene expression by conventional treatment modalities. The understanding of the molecular response of cells and tissues to ionizing radiation has lead to a new appreciation of the exploitable genetic alterations in tumors and the development of treatments combining pharmacological interventions with ionizing radiation that more specifically target either tumor or normal tissue, leading to improvements in efficacy.

## 1. Introduction

Heat shock proteins (HSP) have key roles in cellular stress response and immune modulation. Active areas of interest in the study of HSP and malignancy include their role as prognostic factors, predictors of response to treatment, and their dual role in both tumor cell protection when expressed at high levels within tumors and conversely tumor cell destruction through antigen presentation and processing of tumor-derived HSP-peptide complexes. A growing body of evidence supports the importance of HSP in human cancers in both the intracellular and extracellular environments. Intracellularly, HSP protect cells from proteotoxic stress by a variety of “holding and folding” pathways that prevent the formation of denatured proteins and the progression of lethal aggregation cascades by both necrotic and apoptotic pathways [1,2]. HSP also have a central role in modulation of the immune system, and Hsp72 (HSPA1A/B) can act as an immunological adjuvant [3,4]. Intracellular HSPA1A/B binds processed peptides derived from antigens and shuttles them to the cellular transporter associated with antigen processing (TAP). HSPA1A/B also appears to have additional effects on cytotoxic T-lymphocytes (CTL) that do not require the binding of tumor associated antigens to the HSP. Purified HSPA1A/B induces the activation of CD8^+^ CTL and the secretion of tumor necrosis factor-alpha (TNF-α) and IFN-γ in the absence of peptide loading [5]. The impact of HSP outside the cell has been further revealed in recent years. HSPA1A/B, added exogenously to cells, stimulates the production of pro-inflammatory cytokines TNF-α, interleukin-1 beta (IL-1β) and IL-6 by antigen-presenting cells (APC) [6-8]. This process is referred to as the chaperokine activity of HSPA1A/B. HSPA1A/B appear to play a role in other aspects of non-specific immune responses: HSPA1A/B is found on the cell surface of some tumor cells and is a target of lymphoid activated killer (LAK) cells [9,10]. The majority of studies exploring the role of HSPA1A/B in cellular and immune regulation have been at the pre-clinical level. To date, studies of HSPA1A/B in human malignancies have largely focused on defining the expression of HSPA1A/B in extracted malignant tissue typically obtained at the time of diagnosis [11,12]. Relatively little is known about circulating serum levels of HSPA1A/B [13] and to date the impact of common cancer treatments on circulating HSPA1A/B has been undefined. As characterization of HSP profiles in clinically relevant settings may lead to development of specific new treatment strategies for cancer eradication, the present study was designed to assess the extracellular expression of HSPA1A/B and its potential effect on immune system response in patients undergoing treatment for prostate cancer. Subsequently, *in vivo* and *in vitro* studies were performed to further validate and characterize the clinical findings including the potential for tumor specific immune response and mechanisms for HSP release from intact irradiated tumors.

## 2. Cellular Mechanisms of Heat Shock Response

During evolution, cellular systems have developed mechanisms to adapt to thermal stress and the major target proteins induced by thermal stress are the HSP, which function as chaperones. They are involved in prevention of misaggregation of denatured proteins after stress. HSP also play a role in proper folding of nascent proteins into their required functional conformation. These proteins further regulate the protein turnover and also the cellular redox-state [14]. The best-known HSP are members of the HSP40 (DNAJB1), HSP60 (HSPD1), HSPA1A and HSP90 (HSP90AB1) families. They are highly conserved across prokaryotes and eukaryotes, which points to their importance in cellular protection mechanisms [15]. The mechanisms mediating heat shock response were shown to be not strictly specific for heat only. Interestingly, they are often also cross-protective for other stress factors. In fact, this variation in cellular responses is decisive for using hyperthermia to force cells into apoptotic/necrotic pathways for therapeutic purposes as desired in clinical settings.

Heat-induced HSP expression is mediated by specific promoter sequences, the heat shock elements (HSE). The actual heat-response of these promoters is mediated by the activity of heat-shock factors (HSF), which bind to these HSE sequences and mediate expression of their specific target genes, such as HSP. Three HSF (HSF-1, 2 and 4) have been identified in mammalian cells, which possess additional isoforms for functional variation [16]. Among those, HSF-1 is the key player in heat-mediated gene expression regulation, whereas HSF-2 and HSF-4 activity is not directly linked to heat-responsive regulation of gene expression. In non-heated cells, HSF-1 is localized as a monomer in the cytoplasm and is bound to HSP (e.g. HSPA1A, HSPAB1). During heat activation, HSF-1 is released from HSP and enters the nucleus. In this state of activation, HSF-1 is constitutively hypo-phosphorylated as a monomer. After entering the nucleus, HSF-1 monomers trimerize and are then inducibly hyper-phosphorylated [17]. The phosphorylation status of trimeric HSF-1 is of essential importance for the DNA-binding activity and decisive for the ability to induce transcription [18]. In addition, presence of multiple HSE within a promoter is enhancing heat-inducibility of gene transcription exploited for the construction of heat responsive gene expression vectors in gene therapy. The strong and efficient activation of HSF-1 mediated transcription activation is one major factor that made this conditional system attractive for gene therapeutic uses.

Heat shock induces inhibition of DNA-synthesis and transcription, mRNA-processing and translation, and blocks the progression through cell cycle. The denaturation and misaggregation of proteins is increased leading to enhanced proteasomal and lysosomal degradation [19]. Heat shock leads also to the disruption of cytoskeletal components and changes in membrane permeability with changes in gene expression for increase of cellular thermotolerance [20].

Over-expression of HSPA1As and HSPAB1s in many tumors [21] correlates with the cellular proliferation and survival of oral [22], colorectal [23], and breast cancers [24]. Over-expression of HSPA1A or the less highly conserved HSP27 (HSPB1) protein increases cellular resistance to hyperthermia and other forms of stress by both maintaining normal cellular functions and blocking apoptotic cell death [25]. HSP110 (HSPA4) expression has also been shown to result in a small increase in thermoresistance [26]. Low-level synthesis of HSPA1A can occur in cultured human cells and is also detected in specific tissues from both mice and humans but appears to be upregulated in many cancer cell lines. Regulation of basal level synthesis in human cells is mediated through serum response elements present in the HSPA1A promoter [27], whereas the substantial increase in cellular levels of HSPA1A and other HSP following heat treatment is mediated by HSF-1.

HSPA1A is a highly conserved protein in all organisms, and is composed of three domains, namely N-terminal ATPase domain, substrate-binding domain, and C-terminal lid [28]. It assists in the folding of a large variety of proteins via the association of its substrate binding domain with hydrophobic peptide segment within its substrate proteins in an N-terminal ATPase domain-dependent manner. Furthermore, HSPA1A function is highly regulated by cochaperones through its interactions with the C-terminal lid. HSF mediates the heat shock response by binding to the HSE at the promoter of the *HSP70* gene, and subsequently initiating transcription through formation of active transcription complex assembly [29].

## 3. Heat-Responsive Gene Therapy Systems

Hyperthermia has gained acceptance for cancer therapy of breast carcinoma, colorectal carcinoma, malignant melanoma, head and neck cancer, cervical cancer, esophageal cancer, soft tissue sarcoma, or glioblastoma [30]. A considerable additive or synergistic improvement of therapeutic efficacy can be achieved by combination of chemotherapy, radiation or hyperthermia with gene therapy employed for the regulated expression of cytokine or suicide genes. Hyperthermia ranging from 39 °C up to 48 °C induces transgene expression, representing a promising strategy could be particularly beneficial, which augment the effects of hyperthermia at both cellular and systemic levels. To achieve this, different promoters have been used for construction of viral and nonviral vectors and were tested in numerous gene therapy studies for heat-inducible gene expression [31].

The direct approach for combining hyperthermia with gene therapy is the use of expression vector systems, which employ heat responsive promoters for transgene expression control. The human HSP70B promoter was most frequently used due to low-leakiness and the high-level heat-induced transgene expression, which can be achieved *in vitro* and *in vivo* [32]. For melanoma treatment, heat-induced IL-12 expression with detectable elevated systemic release of the cytokine in association with anti-tumoral efficacy under HSP70B promoter was used [33]. The anti-tumoral effects under HSP70B promoter were further enhanced if heat-responsive IL-12 expression was combined with radiation. The heat shock protein 70 family is a family of multifunctional repair/removal agents for denatured and damaged proteins that can enhance cell survival following injury caused by many different agents including heat, radiation, and chemotherapeutic agents. Basal level cellular expression of inducible HSPB1 is upregulated in many cancer cells and confers a high level of resistance to radiation and chemotherapeutic agents in the absence of heat.

The most prominent developments in this regard are the uses of focused ultrasound (FUS) and of magnetic nanoparticles for temporal and spatial control of heat-responsive gene therapy. Technical improvements of FUS allow the non-invasive and strictly controlled heating of defined tumor volumes [34]. The combination of FUS with magnetic resonance imaging (MRI) allows the real-time monitoring and feedback control of the applied temperature [35].

In an alternative approach, magnetic nanoparticles are used to mediate hyperthermia in the transduced tissue area. For this, either magnetic cationic liposomes containing the magnetic particles and DNA or DNA-loaded magnetic nanoparticles were used. These nanoparticles mediate the heating of the targeted tissue, if alternating magnetic fields are applied. The application of AMF to these tumors caused effective heating of the tissue in association with the induction of the GADD153 promoter-driven TNF-α expression [36]. In a comparable approach, magnetic Mn–Zn ferrite nanoparticles were employed, which had improved capacity for DNA-loading due to their surface modification with cationic polyethylenimide. Intratumoral injection of these nanoparticles and application of AMF led to hyperthermia of 39.5 °C or 42.8 °C in the tumors, which causes efficient heat-induced HSP70B promoter-driven Lac-Z expression. The attractiveness of using nanoparticles lies in the combination of a heat generating system at the cellular level and in its potential as an efficient gene delivery technology.

## 4. Heat Shock and DNA Damage Response

Heat is a pleiotropic damaging physical agent that affects several cellular components to varying degrees; altering protein metabolism, thus affecting the assembly and stability of critical macromolecular complexes. DNA damage inflicted by ionizing radiation triggers the DNA damage response, molecular events that mostly involve the post-translational modification of proteins that activate intracellular signaling pathways [37]. Several cell cycle checkpoints are in place that, on damage such as that inflicted by ionizing radiation, allow the repair, and prevent the propagation, of the damaged DNA. Cells may be halted in their transition from G1 phase to S phase of the cell cycle, intra-S phase and at the G2/M boundary [38]. A defective DNA damage response, in particular a failure to halt the cell cycle, is a feature that is common to many cancers [37]. Some cancer-prone human syndromes arise from defects in specific DNA damage response and DNA repair genes, such as ataxia telangiectasia mutated (*ATM*), nibrin (also known as *NBS1*), *BRCA1* and *BRCA2* [39]. The absence of functional BRCA1 or BRCA2 impairs the ability to repair DSB by HR with additional loss of PARP1 activity, which drives BER and SSBR, then increases the formation of lesions (unrepaired endogenously occurring SSBs) that are repaired by BRCA1-and BRCA2-dependent HR. As a result, cells die from endogenous metabolically induced DNA damage [40]. Oncogene-driven DNA replication stress has been implicated as a cause of constitutive activation of the DNA damage response and tumor progression [39]. Defects in both DNA repair and checkpoint responses in tumor cells affect the response to ionizing radiation and can be exploited for targeted radiosensitization strategies.

Ionizing radiation induces a variety of DNA lesions, including oxidized base damage, abasic sites, single-strand breaks and double-strand breaks. The activity of the DNA repair processes that deal with such damage has long been known to determine the response to ionizing radiation [41]. Deficiencies in pathways that repair double-strand breaks, thought to be the most lethal lesion induced by ionizing radiation, such as non-homologous end joining and homologous recombination, are particularly detrimental to the cells [42]. However, it has become increasingly evident that other repair processes are also needed to ensure genome integrity and survival after ionizing radiation like base excision repair [43], which repairs base damage and single-strand breaks [44] that can be converted to double-strand breaks when encountering a replication fork. Secondary double-strand breaks can also result from repair attempts at complex clustered damage sites that are characteristic of ionizing radiation [45]. These considerations have led to the development of a range of novel compounds that influence DNA repair after chemotherapy and radiotherapy, with the ultimate goal of sensitizing tumor cells to the treatment. Inhibitors of important molecules in double-strand breaks repair, such as ATM or DNA-dependent protein kinase (DNA-PK), have been shown to sensitize cancer cells and xenografted tumors to radiotherapy. The preclinical evaluation of PARP inhibitors has shown that they can increase tumor responses to ionizing radiation in xenograft models. Several PARP inhibitors have already entered clinical trials, and assessments of the combination of PARP inhibitors with radiotherapy are underway [46]. The common overexpression of AP endonuclease (also known as APEX1), which is crucial in base excision repair, and its role in determining radiotherapy and chemotherapy response was the basis of the development of APE inhibitors [47].

Besides DNA repair, cell cycle checkpoints constitute the other important component of the DNA damage response. DNA damage-induced G1/S cell cycle checkpoint activation is almost universally absent in cancer cells, and this absence is frequently caused by mutations in p53 or p53-regulatory processes [48]. However, p53-deficient tumor cells must rely on the S or G2 checkpoints more heavily than normal cells for their survival, which could be exploited therapeutically, because premature mitotic entry would expose the tumor cells to additional damage [49]. The induction of DNA damage by ionizing radiation in normal cells will halt their progression through the cell cycle, preventing further accumulation of damage and its serious consequences.

The ataxia-telangiectasia mutated gene product (ATM) is a protein kinase primarily activated in response to DNA DSBs caused by IR or radiomimetic drugs. Unstressed cells contain inactive ATM in a dimer or higher-order multimer form. Heat is known to induce chromatin alterations [50]. Hyperthermia activates a subset of ataxia-telangiectasia mutated effectors independent of DNA strand breaks and HSPA1A status [51]. Heat treatment before radiation exposure also had no significant effect on chromosomal DNA strand break induction by IR [51]. Heat shock caused a change in the nucleoid halo diameter, but no inhibition of DNA rewinding [51]. In contrast, IR exposure results in a significant inhibition of DNA loop rewinding. Moreover, the nucleoid diameter (which reflects the length of the DNA loops at maximum relaxation) was reduced after heat shock, whereas IR exposure resulted in an increase in nucleoid diameter. Furthermore, Hunt and coworkers did examine whether heat shock induces G_2_-type chromosomal aberrations or inhibited repair of DNA strand breaks or effects both processes [51]. Hunt and coworkers reported that the number of chromosome aberrations detected after irradiation with 0.15 Gy was statistically significant as compared with unirradiated cells [51]. Irradiated cells had detectable levels of chromosomal aberrations after a 0.15 Gy exposure, and no aberrations have been reported in cells that were treated with heat alone [51].

Ionizing radiation also activates the NF-κB pathway through the activation of IκB kinase-α (IKKα; also known as CHUK) as a protective response to damage, and inhibition of this kinase can lead to increased radiosensitvity. Inhibition of the MAPK pathway can also lead to increased radiosensitivity through reduced double-strand breaks repair by both homologous and non-homologous pathways, possibly through the regulation of ATM. The mechanisms by which many of these signal transduction pathways influence radiosensitivity are not completely clear. AKT, MAPK and NF-κB signaling can all inhibit the apoptotic response after DNA damage [52]. The link between the AKT, MAPK and TGF-β pathways and DNA repair have been found, particularly with DSB repair by NHEJ or HR [53]. Because inhibition of these repair pathways causes marked increases in radiosensitivity, this is perhaps the most plausible mechanism that links the signal transduction discussed above to cell kill after irradiation.

Two aspects of the tumor microenvironment have been widely investigated with respect to improving radiotherapy, namely hypoxia (lack of oxygen) and vasculature development. Because hypoxia seldom occurs in normal tissues, it is an attractive tumor-specific target for improving the response to ionizing radiation [54]. Oxygen concentrations of less than 0.02% (0.15 mm Hg) render cells more resistant to killing by ionizing radiation by a factor of 2–3 (ratio of doses under hypoxia versus normoxia to produce equal cell kill) mainly as a result of the radiochemical fixation of DNA damage by molecular oxygen. Hypoxia leads to the activation of the hypoxia-inducible factor (HIF) and unfolded protein response (UPR) pathways, which both determine survival under this stress [55]. High expression of hypoxia-inducible genes is often associated with poor prognosis [56]. Several drugs underwent clinical testing, although the only one to show efficacy in Phase III trials and to remain in routine clinical use is the 5-nitroimadazole nimorazole [57]. The efficacy of such schedules is more at risk from radioresistant hypoxic subpopulations than conventional schedules in which there is more opportunity for the reoxygenation of hypoxic cells during treatment. Recent more extreme hypofractionization schedules are thus likely to benefit most from the use of hypoxic cell radiosensitizers [58].

The compounds have been developed that kill hypoxic cells with far greater efficiency than normoxic cells, which is an alternative to radiosensitizing hypoxic cells and modeling studies indicate that it is the more effective strategy to combine with radiotherapy [59]. New, less toxic drugs are needed, and some tirapazamine analogues are looking promising [60]. Model compounds that reduce hypoxic tolerance have shown efficacy in preclinical models, demonstrating increased ionizing radiation responses resulting from reduced hypoxic fractions [61]. Vasculogenesis also depends on hypoxia, which is more extensive in recurrent tumors, leading to the upregulation of cytokines that in turn recruit and activate the BMDCs that are necessary for vascular formation [62]. In preclinical models, inhibiting vasculogenesis by various interventions, both genetic and pharmacological, dramatically increased tumor responses after radiotherapy and was more effective than inhibiting angiogenesis [63].

## 5. Hyperthermia: Mechanisms and Therapeutic Use

The success of heat-responsive gene therapy greatly depends on whether hyperthermia can be applied efficiently and in a controlled manner to the desired tissue. It is an essential requirement that the applied heat has the temperature level, timely defined duration and spatial control to activate efficiently the heat-responsive vector. To achieve this, already clinically used hyperthermia systems could be employed for local, regional or whole body application of hyperthermia. In analogy to these clinically established technologies, similar and also novel approaches are used for controlled hyperthermic activation of heat-responsive vectors [30]. The first clinical applications of hyperthermia (between 39.5 °C and 43 °C) were performed for treatment of superficious tumor lesions, showing some efficacy [64]. Local hyperthermia has been established for treatment of superficious lesions using specific antennas or applicators to emit micro- or radiowaves for heat generation in the tumor lesion reaching depths of only a few centimeters. Applicators are implanted within the tumor to treat relatively small lesions (b5 cm) for the interstitial and endocavitary hyperthermia. In contrast to these locally restricted hyperthermia treatments, the whole-body hyperthermia are applied for treatment of cancers with distant metastases. In this treatment a maximum temperature of 42 °C can be maintained for 1 h to prevent severe adverse side effects. Improvement in clinical outcome has been shown for tumors of the head and neck, breast, brain, bladder, cervix, rectum, esophagus and for melanoma. In these trials, combination of hyperthermia with radio- or chemotherapy generated best results, due to the sensitizing activity of the applied hyperthermia [65].

### 5.1. Hyperthermia and Hypoxia

Invasion into adjacent tissues and metastasis to distant sites are major features of cancer cells and the cause of 90% of human cancer death [66]. Acquisition of invasive and angiogenic potential is thus critical for metastasis where *hypoxia-inducible factor-1* (HIF-1) pathway plays a pivotal role [67-69]. Hypoxia exists in proliferative tumors and the extent of tumor hypoxia correlates with tumor progression and metastasis [70]. Under hypoxia, HIF-1 α protein is stabilized, dimerized with HIF-1β, and thus can bind to the hypoxia response element to transactivate a battery of genes involved in the promotion of angiogenesis and glucose metabolism to adapt to a stressful environment [71]. The induction of these genes not only gives tumors a survival advantage, but also promotes invasion and angiogenesis [69,71]. Recent studies have suggested that RTK signaling transcriptionally induces HIF-1α [72,73] while hypoxia enhances RTK signaling *via* HIF-1-mediated up-regulation of RTKs [74-76] and the reciprocal relationship between the two distinct oncogenic pathways promotes cancer invasion and angiogenesis [74-76]. HIF-1 transactivates a variety of genes including RTKs [69,74-76], urokinase-type plasminogen activator (uPA) [77], uPA receptor [71,78], MMP2 [71] and VEGF [69], eventually promoting cancer progression. VEGF, the most potent angiogenic factor, stimulates angiogenesis by binding to and activating its cell surface receptor, promoting endothelial cell proliferation and migration. The stability of VEGF receptors depend on HSP90AB1 function and thus HSP90AB1 inhibitors destabilize VEGF receptors [79,80]. Therefore, HSP90AB1 inhibitors efficiently block the proliferation and differentiation of endothelial cells, consequently inhibiting the neovascularization of proliferating tumors.

## 6. Radiotherapy

Approximately 50% of all cancer patients will receive radiotherapy of some form (such as external beam or internal irradiation given as brachytherapy), either alone or in combination with other treatment modalities such as surgery or chemotherapy [81]. For some cancers survival rates after radiotherapy are high (like early stage larynx cancer and non-small-cell lung cancer), whereas for many other cancer sites they are not (e.g., glioblastomas, sarcomas and advanced non-small-cell lung cancer). Accurate delivery of the ionizing radiation dose has greatly improved over the past 2–3 decades, allowing more precise deposition of dose in the tumor while progressively reducing any unwanted dose to surrounding normal tissues [82]. Despite such technical improvements, and despite the fact that radiotherapy is one of the most effective forms of cancer treatment, many patients still suffer from locally recurrent disease after radiotherapy. Clinical factors can explain some of the failures, such as a large tumor and/or advanced tumor stage, which translate into more tumor cells to kill with the same ionizing radiation dose, thus reducing the local control probability. However, many tumors with apparently similar sizes, stages, grades and delivered doses, some will recur and some will not (control rates for many such ‘homogeneous’ populations are not 0% or 100%).

### 6.1. Hyperthermia Enhances Radiation-Induced Cell Killing

Hyperthermia is used to treat external tumors such as sarcoma [83], cervix [84] or with surgically accessible tumors such as prostate [85] and liver [86]. Agarwal and coworkers used multiple approaches to determine the effect of telomerase inactivation on heat- and IR-induced cell killing because telomerase activation has been linked with extension of cell life span. Hyperthermia is a potent radiosensitizer that has been under clinical investigation as a means to improve the response to ionizing radiation (IR)–based cancer treatments, and acts to improve the local tumor control. Recently, the circulating levels of HSPA1A/B in serum in cancer patients are of interest in both defining prognostic significance and identifying a potential target for new therapeutic strategies, such as radiotherapy [87]. Cornford *et al.* assessed the expression of intracellular HSP in tissue obtained from patients with early prostate cancer either at the time of prostatectomy or as an incidental finding at the time of transurethral resection of the prostate (TURP) as well as from patients with advanced disease obtained at the time of TURP [88] and compared with those of control patients with tissue obtained at the time of cystectomy. Immunohistochemical analysis of HSPA1A/B expression revealed similar expression of HSPB1 in early prostate cancers compared with non-neoplastic controls, but diminished expression was noted in morphologically advanced cancers. Plasma HSPA1A/B levels were measured in 125 patients with localized/untreated or hormone refractory prostate cancer and compared with levels for 45 healthy male donor controls of similar age. Plasma HSPA1A/B levels in patients with localized untreated disease were significantly higher than those in the control group. While a primary cutoff point for plasma HSPA1A/B was defined that significantly distinguished the localized untreated patients from the control group, plasma HSPA1A/B was not more effective than PSA as a predictor for diagnosis or stratification of patients into established risk groups [13]. Our group further assessed the impact of common treatments for prostate cancer on serum HSPA1A/B levels and to characterize its mechanism of release and biological significance using Mouse orthotopic xenograft of human prostate cancer. There was no increase with AST, but a significant increase in circulating serum HSPA1A/B was noted in response to radiotherapy. Also, there was a significant increase in cells phenotypically characterized at CD8^+^ cytotoxic T lymphocytes and CD56^+^ natural killer (NK) cells and a concomitant increase in the pro-inflammatory cytokines IL-6 and TNF-α. Zitvogel *et al.*, showed that exosomes produced by mouse DC pulsed with tumor peptides induce the rejection of established tumors in an antigen specific, T cell-dependent fashion in which the anti-tumor effects were associated with long-term survival [89]. Indeed, this property of exosomes is currently being assessed for its potential as a cancer vaccine in phase I clinical trials [90]. Our group hypothesized that radiotherapy stimulates the passive and active release of HSPA1A/B from tumors. Passive release is achieved by the direct radiotherapy-induced necrosis of tumors. This liberates heat shock protein peptide complexes (HSP-PC) which bind to and stimulate APC to produce pro-inflammatory cytokines, chemokines and reactive oxygen species, increase the expression of costimulatory molecules and augments the maturation of dendritic cells, a process known as the chaperokine activity of HSPA1A/B found in the extracellular milieu [91-93]. Recently, it was demonstrated that HSPA1A/B can also be induced by an active process [94]. Consistent with our findings in prostate cancer patients, radiation exposure resulted in a significant increase in serum HSPA1A/B concentrations in both human xenografts and syngeneic tumor-bearing mice. The dose-dependent nature of HSPA1A/B levels in response to radiation directly results in maximum release of HSPA1A/B into the serum by 24 hours post-exposure and returned to baseline values by 96 hours in both human xenografts and syngeneic tumor-bearing mice. Also, we demonstrated that HSPA1A/B was released into circulation in response to irradiation in a similar fashion as HSPA1A/B, *albeit* to lesser levels in DU-145 human xenograft as compared to PC-3 human xenograft. Interestingly, the kinetics of HSPA1A/B release induced by gamma irradiation peaked at 48 hours post exposure, whereas HSPA1A/B peaked at 24 hours in both PC-3 and DU-145 prostate cancer cells, which is indicative of a difference in the mechanism by which HSPA1A/B is transported within the cell and subsequently released into the extracellular milieu, as compared with HSPA1A/B.

Ionizing radiation as applied in the radiotherapy clinic induces a complex response in cells. Some processes aim to repair damage, whereas others counteract propagation of the damage or induce cell death. Tumor-targeted radiosensitization that partly depends on impaired DNA repair processes that result from mutated DNA repair genes within tumors. Hypoxia down regulates the expression of repair proteins and affects HR [95]. The repair of DNA damage is crucial to genomic integrity, and deficiencies in repair are known to have a large influence on cellular survival after ionizing radiation. More recently, it has also been shown that aberrantly activated signal transduction pathways in cancer cells can influence cellular radiosensitivity. Finally, the damage caused by ionizing radiation is markedly affected by oxygen levels, and irradiation under hypoxic conditions, as occurs in most tumors, reduces cell kill.

### 6.2. Radiation-Induced Damage

Data on cardiac complications after radiotherapy for the loco-regional management of breast cancer are distinguished from many other data sets as many breast cancer patients have been treated in clinical trials enabling systematic investigations over many decades. The results of these analyses prove radiation-induced cardiac complications on the one hand, while on the other they reflect the improvements in radiotherapy planning and delivery during these years, which greatly contributed to avoidance of the heart and diminished the risk of side effects. Cardiac toxicity has been implicated as the primary cause of excess non-breast cancer mortality in early breast cancer radiotherapy studies. An estimate of the aggregate incidence of radiation-induced cardiac disease is between 10% and 30% by 5–10 years after treatment. Up to 88% of patients have asymptomatic abnormalities. Until recently, the cardiovascular system has been considered as relatively radio-resistant. In radiotherapy, the currently recommended tolerance dose for the heart is 40 Gy in fractions of 5 × 2 Gy/week if the whole organ is irradiated (risk of pericarditis). If only parts of the heart are exposed, even higher tolerance doses are accepted, *i.e.*, 45 Gy if a volume of 66% is affected and up to 60 Gy if a volume of 33% is irradiated [96]. Epidemiological findings even indicate that the heart might be one of the most critical dose-limiting organs in radiotherapy.

Different groups of people have been exposed to low doses (<1 Gy) of radiation to the heart. Among these are nuclear industry workers [97] and patients receiving radiation for diagnostic purposes. Radiologists and other medical workers received relevant occupational doses of radiation in the first half of the 20th century, when radiation protection measures were given only scant consideration [98]. Awareness of the potential risk of late cardiovascular disease after exposure to low radiation doses was initiated by a recent analysis of mortality from cancer and nonmalignant diseases among atomic bomb survivors in Japan. These individuals received one whole-body exposure. High-dose radiation to the heart can damage all cardiac structures and peripheral vessels with variable onset. All these forms of damage differ with regard to their probability after radiation exposure, latency and clinical features and lead to distinct histopathological changes. Often, several cardiac structures are affected, such that combinations of conditions occur. Pericardial damage leading to fibrous thickening and fluid production is one of the most frequently described forms of damage. Pericarditis may occur with a latency of months or years if large volumes of the heart receive doses of more than 40 Gy. Most cases start as asymptomatic exudative pericarditis, which progresses in approximately 20% of patients who develop either constrictive pericarditis or cardiac tamponade with hemodynamic compromise. The incidence of pericarditis has decreased with improvements in the conformality of dose distribution. Damage to the myocardium causes myocarditis, which can lead to progressive diastolic dysfunction and restrictive hemodynamics (≥1 year after irradiation), ending in congestive heart failure. These clinical complications correlate with diffuse interstitial fibrosis and microcirculatory damage leading to capillary obstruction or extensive fibrosis. Symptomatic cardiomyopathy during or shortly after radiotherapy is only seen in combination with anthracycline chemotherapy.

Damage to the vascular system can trigger arteritis with premature coronary artery disease and accelerated atherosclerosis, which leads to ostial and proximal stenosis of the coronary arteries, which is characteristic of radiation-induced coronary artery disease. Vascular lesions correspond to intima hyperplasia and lumen wall collagen deposition, and finally atherosclerosis can lead to (fatal) cardiac infarction. Damage to vessels can also pertain to pulmonary vessels leading to pulmonary hypertension. The risk for coronary heart disease seems to be increased by doses as low as 1–2 Gy to the whole organ, especially if delivered as whole-body exposure. Seldom sequelae are endocardial and valvular damage progresses over time with stenosis and regurgitation. Pathological correlates are valvular cusp and/or leaflet fibrosis. The development of valve disease is similar to the general population regarding hemodynamics, natural history, and progression. Autonomic dysfunction leads to supraventricular tachycardia or heart rate variability. The frequency of conduction abnormalities in asymptomatic cancer survivors attributable to radiation only is unknown since causality is difficult to investigate.

Systematic morphometric studies in rats show that capillary volume and length density begin to decline approxi–mately 20 days after heart irradiation, and that the decline continues in a dose-dependent manner. In rats, myocardial cell death, reduced myocardial density, and degeneration occur 10–20 weeks after irradiation, coincident with early signs of decreased cardiac function. The relevance of these mechanisms is supported by clinical studies showing regional perfusion defects in breast cancer patients between 6 months and 5 years after irradiation, which is related to the volume of the left ventricle included in the irradiated field. In the case of capillary rarefaction, there is a loss of the endothelial cell marker enzyme, alkaline phosphatase increases with time, which is involved in regulating endothelial cell proliferation and microvascular blood flow by dephosphorylating extracellular nucleoside phosphates. Ultrastructural studies showed that the enzyme loss was not caused by a loss of endothelial cells but associated with signs of endothelial cell activation, such as swelling, lymphocyte adhesion, and extravasation. In larger vessels, radiation induces oxidative stress in the endothelium, leading to accelerated development of intimal thickening and the formation of vulnerable inflammatory atherosclerotic plaques leading to monocyte adhesion and transmigration into the subendothelial space.

Chronic inflammation is a major pathophysiological factor contributing to the development of atherosclerosis [99]. In atomic bomb survivors, C-reactive protein, interleukin 6, tumor necrosis factor α, and interferon-γ increased with radiation dose, which results suggest significantly increased inflammatory activity after whole-body exposure, which might partially explain the increased incidence of ischemic heart disease in atomic bomb survivors despite the low dose to the heart. Anti-inflammatory and anti-coagulant therapies were less effective at inhibiting radiation-induced atherosclerosis than age-related atherosclerosis, suggesting a more complex mechanism for lesion development after irradiation. One approach is to inject mesenchymal stem cells overexpressing hepatocyte growth factor (HGF) or a replication-deficient adenovirus carrying the HGF gene to stimulate the regeneration of cardiomyocytes. A further treatment approach might be statins, which are used to treat spontaneous heart disease by reducing the production of cholesterol. In this indication, statins reduce inflammation, slow the formation of new atherosclerotic plaques, occasionally reduce the size of existing plaques, stabilize plaques and make them less prone to rupturing and forming clots.

### 6.3. Differential Effects of Lethal and Non-lethal Temperatures

Hyperthermia can be used by itself and results in impressive shrinkage and even complete eradication (10-15%) of tumors, but usually do not last and the tumors regrow. Combined hyperthermia and radiation has been reported to yield higher complete and durable responses than radiation alone in superficial tumors. Hyperthermia is one way to overcome the radio resistance of tumor cells. It is possible to combine hyperthermia safely with further low-dose radiation in the situation where a radical dose has already been delivered. In addition, there seems to be evidence that whole body hyperthermia provides a measure of protection against radiation-induced thrombocytopenia. Hyperthermia improves the therapeutic index of TBI (total body irradiation), not only by increased neoplastic cell kill, but also by inhibiting the expression of radiation-induced damage to the normal cell population. Some agents not cytotoxic at normal temperature show cell killing abilities at higher temperatures: alcohols, amphotericin B, cysteine, and cysteamine. The new agents interferon, TNF and lonidamine and some hypoxic cell sensitizers are all potentates by heat. Hyperthermia can augment the cytotoxicity without increasing myelosuppression, and reverse drug resistance to chemotherapeutic agents. It has been shown in several studies that the use of hyperthermia can enhance the delivery of monoclonal antibodies to tumors with resultant improvement in anti-tumor effects. The spread into tissues of liposome-carried chemo drugs increases considerably compared to that under normal temperature. Hyperthermia is also an immune system enhancer, and very effective in providing pain relief, controlling bleeding, and useful in other conditions such as prostatic hypertrophy and psoriasis.

Hyperthermia side effects for the external methods include pain, unpleasant sensations and burns in a small percentage of patients. In the case of the internal pyrogens, which are sometimes bacterial toxins, the situation is more complicated as bacterial toxins can induce serious, even fatal reactions in humans, depending on dosage. Ultrasound hyperthermia in areas where the tumor is over a bone will cause bone pain. Whole body hyperthermia can result in neuropathies. Extracorporeal systemic hyperthermia, where the blood is routed from the body as in dialysis has two advantage: higher possible temperatures and more homogeneous heating. The side effects, however, have been considerable with frequent persistent peripheral neuropathies, abnormal (and sometimes lethal) blood coagulation, some damage to liver and kidneys, and brain hemorrhaging and seizures. Hyperthermia should be administered to patients who are awake and can report any problems as they experience them. The clinical use of moderate hyperthermia in combination with ionizing radiation is thought to benefit from the complementary cell-cycle sensitivity of these two modalities because radioresistant S-phase cells are extremely heat-sensitive [100]. Specifically, increased cell-cycle delays in mid to late S/G2 phases were found to be associated with increased G2/M checkpoint abrogation, which leads to inappropriate mitotic entry. Further, subsequent to delays in late S and G2 phases following X-irradiation, G2/M checkpoint abrogation was observed which was correlated with four to sixfold increase in cyclin B1 content per cell [101]. Previous reports of moderate hyperthermic studies of mitotic catastrophe and decreases in clonogenic survival have implicated with lethality. For example, a 12h exposure of S-phase CHO cells to 41.5 °C hyperthermia lead to a 50% incidence of mitotic catastrophe and a corresponding decrease in survival [102] and chronic exposure of HeLa cells to 41.5 °C produced a yield of spontaneous premature chromosome condensation (SPCC) followed by nuclear fragmentation, which showed a one-to-one correlation with cell killing [103]. Therefore, 4h, 41.5 °C (non-lethal) exposure delivered prior to X-irradiation produced maximal radiosenstization [104], yet no studies have demonstrated a causal role for SPCC in lethality under moderate hyperthermia.

An optimization treatment planning model was designed that specifies the most appropriate laser parameters to permit complete tumor destruction by maximizing injury and eliminating HSP expression in the tumor. The model also permits preservation of the healthy surrounding tissue by minimizing the tissue injury and enhancing recovery by induction of HSP expression at 43.8 °C in the tumor region and 42.8 °C in the healthy tissue [105,106]. Extended heating durations of 30 minutes are required to induce sufficient thermal injury for these therapies. The equivalent thermal dose of 48.8 °C for 1 min enabled comparison with the HSP and injury fraction based optimization. The temperature-based optimization yielded an insufficient amount of thermal injury and high levels of HSP expression in the tumor. Without imposing more stringent constraints and objective functions based on desired thermal injury fraction and HSP expression, the lack of thermal injury and elevated HSP expression in the tumor is certain to result in tumor recurrence and resistance to subsequent chemotherapy and radiation treatments.

Implementation of both HSP expression and injury fraction objective functions based on cellular and tissue data in the optimization process permitted successful selection of truly optimal therapies with maximum injury and elimination of HSP expression in the tumor and minimum injury and HSP expression elevation in the healthy tissue. The temperature prediction provided by the Penne's equation corresponds closely with measured data using Magnetic Resonance Thermometry during laser irradiation of prostate tumors [105,106]. The steepest descent method for optimization was capable of determining effective laser parameters based on specified objective functions developed from strict criteria related to the desired tissue response. Alternate optimization strategies such as Newton's or quasi-Newton's method could deliver a better convergence rate, however, its utilization will provide minimal improvement due to the existing efficiency of the adaptive finite element algorithm which reaches convergence within very few steps and requires minimal CPU time. Incorporating appropriate thermal and optical properties for the tissue of interest for the temperatures and wavelengths considered is critical to achieving accurate prediction and optimization of the tissue response to laser therapy. Currently, the model does not incorporate the dynamic optical properties associated with denaturation of proteins during the laser heating process, which may lead to alterations in tissue absorption and scattering properties.

### 6.4. Strategies to Overcome Radiation-Induced Damage

Modern targeted radiotherapy aims to cause maximum killing of cancer cells while minimizing damage to normal tissue. However, these treatments are not without side effects, and cancer survivors are still at an increased risk of developing a range of unpleasant, and even life-threatening, side effects. These side effects are caused by a combination of normal cell killing (both parenchymal and vascular cells) and stimulation of inflammatory, thrombotic and fibrogenic processes. Side effects may develop during or shortly after a course of fractionated radiotherapy (e.g., mouth ulcers or dry mouth after radiotherapy for head and neck cancer) or many months to years after the end of treatment (e.g., frozen shoulder edema and fibrosis or heart failure after radiotherapy for breast cancer, or cognitive defects after irradiation of brain metastases). Modifiers of normal tissue responses to ionizing radiation can be classified as prophylactic (radioprotectors), mitigators or therapeutic agents. Prophylactic agents, given before ionizing radiation exposure, include free radical scavengers that prevent the fixation of the initial radiochemical event, inhibitors of p53-induced early apoptotic cell death and antioxidants that inhibit early inflammatory reactions. Mitigators are given during or shortly after radiotherapy, before clinical presentation of ionizing radiation injury. They include antioxidants, growth factors and stem cell- or progenitor cell-based approaches to stimulate proliferative regeneration and support the survival and differentiation of healthy normal cells. Strategies for reducing the severity of acute ionizing radiation reactions aim to either prevent initial cell death or stimulate the regeneration of damaged tissues. Radical scavengers are effective when given before irradiation and, as they react with free radicals in competition with oxygen, the degree of radioprotection is highly dependent on oxygen tension, being maximal at intermediate oxygenation [107]. Amifostine has emerged as the best radical scavenging radioprotector in terms of efficacy to toxicity ratio and it has been widely tested in the clinic, particularly for reducing the incidence of xerostomia in patients with head and neck cancer. Protection against ionizing radiation-induced pneumonitis has also been reported in some clinical trials in patients with lung cancer [108].

Haematopoietic growth factors have been used for many years to stimulate the recovery of the bone marrow and to prevent chemotherapy or total body irradiation (TBI)-induced neutropenia after myeloablative conditioning for stem cell transplantation. This approach is now being extended to non-haematopoietic growth factors, such as keratinocyte growth factor (KGF), and preclinical studies have shown substantial protection against ionizing radiation-induced oral mucositis [109], intestinal damage [110] and pneumonitis [111]. Transient inhibition of p53 can be an effective strategy for protection against acute ionizing radiation injury in specific epithelial and lymphoid tissues, through the direct inhibition of apoptosis in the relevant stem cell compartments. p53 has an essential role in mediating cell cycle arrest and apoptosis in response to gentoxic insults, thus preventing the replication of cells with damaged DNA and inhibiting tumorigenesis. However, p53-dependent apoptosis of stem cells also results in acute ionizing radiation injury in several normal tissues including the bone marrow, intestine and testes. Transient inhibition of p53 (by genetic manipulation or small-molecule inhibitors) has been shown to provide effective protection against bone marrow and intestinal apoptosis after TBI of mice, without promoting tumor formation or influencing the ionizing radiation sensitivity of tumors [112]. Mesenchymal stem cells (MSC) have extensive proliferative capacity and they can be selected from the bone marrow (on the basis of specific cell surface markers) and expanded *in vitro* before transplantation. Systemically delivered MSC have been shown to engraft in irradiated skin, esophagus and intestine and to stimulate tissue regeneration and to reduce the severity of ionizing radiation damage [113]. Although stem cells have now been isolated and expanded from several normal tissues, the salivary gland is, so far, the only tissue in which transplanted adult stem cells have been shown to both localize to the irradiated tissue and to lead to an improvement of tissue structure and function [114]. One earlier study found that transplantation of immortalized embryonic neural stem cells delayed the onset of ionizing radiation-induced paralysis after cervical cord irradiation [115].

Vascular damage is one of the major underlying causes of late ionizing radiation injury. In small vessels endothelial cell damage initiates inflammatory and coagulation cascades, which lead to vascular leakage, micro-thrombus formation and secondary tissue ischemia. Overproduction of inflammatory cytokines, such as TGF-β and thrombin, also drives smooth muscle cell proliferation and collagen production in fibroblasts, initiating the development of fibrosis. Restoration of the thrombo– hemorrhagic balance in endothelial cells [116] and the inhibition of ionizing radiation-induced activation of TGF-β [117]. Animal studies have shown that bone marrow-derived dendritic cells (BMDC) efficiently engraft to ionizing radiation-damaged tissue and contribute to tissue repair and improve the function of the esophagus [113] and salivary gland [118]. Only very limited incorporation and clonal expansion of mobilized or transplanted cells into the irradiated gland was seen, although newly formed blood vessels did contain bone marrow-derived dendritic cells. Because of the paracrine effects, such as secreted growth factors and cytokines, stimulated vascular regeneration and the proliferation of surviving stem cells thus contributed to reduced damage [118].

## 7. Current and Future Radiation Oncology Strategies

One further direction to reduce dosage to the heart in selected indications might also be radiotherapy with particles such as carbon or helium ions and protons. Charged particles have different depth dose distributions as opposed to photons, depositing most of their energy at the very end of their trajectory in tissue, which results in a very sharp and localized peak of dose (Bragg peak). Compared to advanced photon techniques, radiotherapy with particles therefore offers potential, especially where (large) geometrically complex target volumes are located in close proximity to organs at risk and necessitating steep dose fall-off. One example of cardiac sparing by means of proton radiotherapy in dosimetric studies is treatment of the breast including regional nodes [119]. For centrally or superiorly located stage I non-small cell lung cancer (NSCLC), proton therapy, in particular intensity-modulated proton therapy (IMPT), can deliver ablative doses to the target volume and significantly reduce doses to normal tissues (heart, aorta, pulmonary vessels, lung, brachial plexus, spinal cord) compared with photon stereotactic body radiation therapy [120]. Compared with intensity modulated photon radiotherapy, IMPT spared more lung, heart, spinal cord, and esophagus, even with a dose escalation from 63 Gy to 83.5 Gy [121].

The prevalence of cardiac complications will realistically decrease in patients treated recently. Radiation-induced late complications in organs at risk can obviously be reduced rather than avoided by diminishing radiation dose to these organs. To this end, continuous efforts are aimed at minimizing the dose delivered to organs at risk while maintaining or even maximizing the dose to the target. Recent but widely implemented techniques contributing greatly to conformal dose distribution, particularly in the thorax, include image guidance (IGRT), e.g., using kV imaging and cone beam computed tomography to reduce set-up errors and intensity-modulated photon radiotherapy (IMRT) [122]. IMRT can improve conformality compared with conformal 3D techniques, especially in cases of geometrically complex targets. However, multifield IMRT produces a bath of low doses to greater volumes of normal tissues. Consequently, concern exists about the appraisal of IMRT in terms of avoiding cardiac complications.

## 8. Conclusions

Hyperthermia treatment has shown promising results in the treatment of cancer patients. Heat does not induce DNA double-strand breaks but rather appears to inhibit repair of DNA damage especially in the case of IR-induced DNA damage, which produces complex closely clustered damage on opposed DNA strands, heat shock pre-irradiation results in delays in completing multi-step repair processes. Heat may also cause protein aggregates to sequester the proteins involved in DNA damage repair. Heat shock proteins modulate the effects of heat by binding unfolded protein domains and maintaining the protein in a soluble form until it can be refolded or degraded, thereby minimizing protein aggregation. Moreover, HSP have been shown to enhance the function of DNA repair enzymes such as human apurinic/apyrimidinic endonuclease. Clinical studies have shown that hyperthermia can directly kill tumor cells and can act as a sensitizer for radiation therapy or chemotherapy. Hyperthermia is a potent radiosensitizer that has been under clinical investigation as a means to improve the response to IR–based cancer treatments that acts to improve the local tumor control. Hyperthermia itself has several cellular effects that should be synergistic with IR-induced tumor cell killing. Unlike the IR response, neither hypoxic nor plateau-phase cells are resistant to heat-induced cell killing. Clinical trials have shown significant benefits from adding hyperthermia to radiotherapy regimens for a number of malignancies and understanding the mechanisms involved in heat-mediated IR sensitization has become clinically important. Therefore, targeting a combination of tumor specific and DNA repair pathways will not only enhance heat-induced radiosensitization, but will also decrease the overall level of normal tissue toxicity occurring during radiotherapy, which could eventually help to improve sequencing of the heat and IR treatment to obtain a better clinical outcome. The combination of specificity and efficacy of an approach can be considered as a measure of its merit. Attacking or exploiting hypoxia is highly tumor specific, because hypoxia occurs almost universally in solid tumors and is rare in normal tissues. Increasing cellular radiosensitivity is not tumor specific, because DNA repair and signal transduction pathways that influence radiosensitivity also operate, and are often essential, for normal tissue survival and function. Modulating cell cycle checkpoints can be regarded as another approach for increasing tumor cell radiosensitivity that is partially tumor specific because, as discussed above, most tumors have a defective G1/S checkpoint, rendering them more susceptible than normal tissues to the inhibition of the remaining checkpoints. Approaches to protect normal tissues by reducing sensitivity to ionizing radiation may be risky unless there is good specificity for non-malignant cells. It will be very interesting to give hyperthermia and irradiation simultaneously, but there is no equipment available to date.
